# Psychological Interventions for Patients with Coronary Heart Disease and Their Partners: A Systematic Review

**DOI:** 10.1371/journal.pone.0073459

**Published:** 2013-09-05

**Authors:** Jane Reid, Chantal F. Ski, David R. Thompson

**Affiliations:** Cardiovascular Research Centre, Australian Catholic University, Melbourne, Victoria, Australia; Iran University of Medical Sciences, Islamic Republic of Iran

## Abstract

**Objectives:**

Despite evidence that patients with coronary heart disease (CHD) and their partners report significant psychological distress, and suggestions that involving partners in interventions alleviates such distress, no systematic reviews have examined this. The objective of this study was to systematically review evidence on the effectiveness of psychological interventions for patients with CHD and their partners.

**Methods:**

CENTRAL, Medline, EMBASE, CINAHL and PsycINFO databases were searched through October 2012. Randomized controlled trials evaluating psychological interventions for patients with CHD and their partners were included. Selection of studies, study appraisal, data extraction and analysis were undertaken using standard methods.

**Results:**

Seven studies comprising 673 dyads (patient and partner) were included. Psychological interventions result in modest improvements in patients' health-related quality of life, blood pressure, knowledge of disease and treatment, and satisfaction with care, and in partners' anxiety, knowledge and satisfaction. There was a non-significant trend for improvements in anxiety for patients, and depressive symptoms for both patients and partners. There was no evidence of a significant effect on mortality, morbidity or other cardiovascular risk factors for patients, or social support for patients and partners.

**Conclusions:**

Psychological interventions for patients with CHD and their partners were found to improve health-related quality of life, blood pressure, knowledge, and satisfaction with care for patients, and anxiety, knowledge, and satisfaction with care for partners. However, as the overall quality of the evidence was low, these results should be interpreted with caution.

## Introduction

Coronary heart disease (CHD), particularly myocardial infarction (MI), imposes a significant physical, psychological and social burden on patients and on their family members and commonly elicits anxiety and depression. The prevalence of major depression in patients after MI has been estimated to be around 20% and of depressive symptoms between 7% and 31% [1]; the prevalence of anxiety has been estimated at 30% to 40% [2].

Both depression and anxiety are associated with worse prognosis after a cardiac event. People with depression and CHD have an estimated 1.5 to two times increased risk of adverse cardiovascular outcomes, including mortality and new cardiovascular events [3]–[5], and people with anxiety and CHD have an estimated 36% increased risk of adverse cardiac outcomes [2]. In addition, depression and/or anxiety in people with CHD is associated with adverse quality of life [6], [7].

The patient's family, in particular the partner, also commonly experience psychological distress. Levels of depression and anxiety for the partner have not been studied as extensively as for the patient, but it has been suggested that the degree of psychological distress is at least as great as the patient's [8]. Considering the potential impact of CHD on the patient-partner relationship, the response and coping strategies of patient and partner play an integral role in determining adjustment and functioning. It has been recommended that the patient and partner should be conceptualised as a dyadic unit and therefore both should be involved in care after the event [8].

Psychological interventions aim to assist with the adjustment process after the cardiac event, thus reducing depression and anxiety and improving quality of life. Systematic reviews of psychological interventions for patients with CHD [9]–[11] report beneficial effects on depression and anxiety but inconsistent effects on mortality, new cardiac events and quality of life.

Systematic reviews of the effectiveness of psychological interventions for patients with chronic diseases, such as cancer and arthritis, as well as CHD, and their partner or family member [12]–[14] report potential benefits for both the patient and family member (predominately the partner). The potential benefits for the patient included improvements in mental health, physical health (including mortality) and quality of life; and for the partner improvements in mental health and quality of life. Significant clinical heterogeneity, however, was present with respect to patients' diagnoses, family members involved, interventions utilized, and how outcomes were measured. In addition, there was only minimal reporting of results for CHD patients and their partners. A recent systematic review of psychological interventions for patients with CHD [11], which undertook meta-regression analyses, found that family interventions were less effective than non-family interventions; however this review only assessed patient outcomes.

Given the adverse impact of psychological distress and CHD it is important to reduce the prevalence and severity of depression and anxiety in patients with CHD. Psychological interventions appear effective in providing such benefit. Involving the partner in the intervention may provide additional benefit for both the patient and the partner, but to date no systematic review to examine this has been reported.

The aim of this systematic review was to evaluate the effectiveness of psychological interventions for patients with CHD and their partners.

## Methods

The following criteria were used for considering studies for this review:

### Types of studies

Randomized controlled trials with any length of follow-up, available as full trial report, were eligible for inclusion. There were no language restrictions.

### Types of participants

Trials which included adults, 18 years of age or older, with CHD were included. CHD was defined as a primary diagnosis of myocardial infarction (MI); angina; or revascularization procedures such as percutaneous coronary intervention (PCI), coronary artery bypass grafting (CABG); or angiographically confirmed CHD. Studies that included mixed participant groups were included if the results were reported separately for CHD patients, or if more than 80% of the participants had CHD. Studies were excluded which only included participants with heart failure.

### Types of interventions

To assess the effectiveness of psychological interventions, only trials which compared the intervention group to a control group receiving usual care were eligible.

Psychological interventions were defined as all types of counselling, psycho-education, social support or therapy aimed at improving general well being (for example mental health and quality of life). The intervention could include education as long as this was provided with a psychological component. Other interventions, such as medication and/or exercise, could be included as long as both/all groups received the additional intervention(s). The intervention could be of any duration, any format (group or individual) and could be delivered by any health care workers (for example nurses, psychologists, physicians or social workers). Studies which utilized an intervention which was education or relaxation only were excluded.

The intervention needed to include the patient's partner (defined as support person – spouse/partner, primary carer or other support person (e.g. friend)) and at least 50% of sessions had to be attended by the partner. Trials which included some partners were included if the results are reported separately for our group of interest, or if more than 80% of the partners participated. Studies were excluded if the participant's support person only included co-workers.

### Types of outcome measures

Primary outcomes for patient and partner, measured using a validated instrument, were:

1. depression
2. anxiety
3. health-related quality of life

Secondary outcomes for patient were:

1. mortality (cardiac and all-cause)
2. cardiovascular morbidity (MI, stroke, revascularisations (PCI/CABG))
3. cardiovascular risk factors

Secondary outcomes for patient and partner were:

1. social support
2. knowledge of disease and treatment
3. satisfaction with care

## Search Methods for Identification of Studies

### Electronic searches

The following electronic bibliographic databases were searched in October 2012:

- Cochrane Central Register of Controlled Trials (inception to present)
- MEDLINE (on Ovid) (inception to present)
- MEDLINE (on Ovid) In-Process & Other Non-Indexed Citations
- EMBASE (inception to present)
- CINAHLPlus (on EBSCO) (inception to present)
- PsycINFO (on Ovid) (1967 to present)

Search terms used were a combination of subject headings and key words; these were related to CHD, psychological terms, partner/family and RCT. The following search strategy was used for Medline and was adapted for other databases:

((cardiovascular disease/ OR Heart Diseases/ OR exp Myocardial Ischemia/ OR exp Myocardial Revascularization/ OR coronary.mp. and exp Stents/ OR acute coronary syndrome*.tw. OR heart disease*.tw. OR (heart adj3 surg*).tw. OR (Myocardi* and (infarct* or ischemi* or ischaemi*)).tw. OR (coronary and (disease* or artery or arteries or stent* or angioplast* or bypass or by-pass or intervention*)).tw. OR (cardiac and (disease* or surg* or bypass or by-pass or intervention*)).tw. OR angina.tw. OR exp Myocardial Revascularization/ OR CABG*.tw. OR PCI*.tw. OR PTCA*.tw.) AND exp Counseling/ OR exp Psychotherapy/ OR Counseling.tw. OR counselling.tw. OR (Psycholo* or Psychotherap* or Psycho-therap* or Psychosocial* or Psycho-social* or psychoeducation* or psycho-education*).tw. OR depression/ OR stress, psychological/ OR Anxiety/ OR (Depress* or Anxiety or stress).tw. AND (couples therapy/ or family therapy/ or marital therapy/ OR family/ or family relations/ or family conflict/ OR Caregivers/ OR (Famil* or Spous* or Partner* or next-of-kin or couple* or marital).tw. OR significant other.tw.) AND (randomized controlled trial.pt. OR controlled clinical trial.pt. OR randomi?ed.tw. OR placebo.ab. OR clinical trials as topic.sh. OR randomly.ab. OR trial.ti.)) NOT (exp animals/not humans.sh.)

The WHO International Clinical Trials Registry Platform (ICTRP) was searched in October 2012. The search strategy used was: (coronary OR cardiac OR heart) in condition AND (Counseling OR Psychol* OR Psychosocial) in intervention.

### Searching other resources

Reference lists of eligible trials and relevant systematic reviews were searched for additional studies. Scopus was used to search for relevant articles/studies which cited included trials and relevant systematic reviews.

## Data Collection and Analysis

### Selection of studies

Studies were excluded based on titles and abstracts, and full text articles were retrieved and reviewed as necessary. Studies were assessed for inclusion in the review using a pre-designed eligibility form based on the inclusion criteria. If a trial did not contain sufficient information for a decision to be made about its eligibility, further information was sought from the trial's authors. The two authors independently determined which studies met the selection criteria. Disagreements about study eligibility were resolved by discussion, or with consultation of a third reviewer.

### Data extraction and management

For each included trial, two authors independently extracted data using a pre-designed data extraction form. Data were extracted which described the characteristics of the trial (country conducted, when conducted, setting, design, patients randomized and duration of follow-up); the participants (age, gender and diagnosis); and the intervention (significant other, personnel conducting the intervention, type of intervention, format and duration). The comparator used in the study was also documented. Relevant outcomes were also extracted. If the reported data were incomplete or unclear, study authors were contacted. The reviewers worked independently, and disagreements were resolved through consensus, or in consultation with a third reviewer.

### Assessment of risk of bias in included studies

Two of the review authors independently used The Cochrane Collaboration's tool for assessing risk of bias [15]. The following domains were assessed as ‘low’, ‘unclear’ or ‘high’ risk of bias:

1. sequence generation;
2. allocation concealment;
3. blinding of outcomes assessment;
4. incomplete outcome data;
5. selective outcome reporting;
6. other sources of bias (e.g. conflict of interest).

The domain ‘Blinding of participants and personnel’ was not applicable for this review.

The gradings between reviewers were compared and any differences were resolved by discussion or in consultation with a third reviewer.

### Measures of treatment effect

The treatment effect was analysed using relative risks (RRs) for dichotomous outcomes; and mean difference (MD) or standardized mean difference (SMD) for continuous data. Ninety-five percent confidence intervals (CI) were calculated for all analyses. All statistical analyses were undertaken using Review Manager Version 5.1 [16].

### Unit of analysis issues

For trials with more than one intervention group, or more than one control group, all eligible intervention groups were combined and all eligible control groups were combined to create a single pair-wise comparison, using methods described in the Cochrane handbook [15]. Groups were deemed eligible if they met the review's selection criteria.

For trials which utilized a factorial design, data were combined from intervention groups of interest and control groups of interest regardless of other intervention(s) being evaluated. As stated in the criteria for considering studies (section above), the other treatment(s) needed to apply to both intervention and comparison groups for the trial to be eligible.

### Dealing with duplicate publications

When more than one publication of an original trial was identified, the articles were assessed together to maximise data collection. Outcomes were obtained from the longest follow-up period.

### Assessment of heterogeneity

Reviewers first assessed statistical heterogeneity by visual examination of the generated forest plots based on whether there was CI overlap and whether direction of treatment effect was consistent across studies.

Statistical heterogeneity was assessed using the Q test (significant if p<0.1) and I^2^ statistic; I^2^ statistic greater than 50% may represent substantial heterogeneity [15] and reasons for heterogeneity were examined.

### Assessment of reporting biases

Although this review did not specifically exclude unpublished studies, it was possible that unpublished studies (e.g. from the grey literature) were less likely to be identified. The WHO International Clinical Trials Registry was searched to determine any evidence of the existence of reporting bias, such as if a trial was registered but not published.

### Data synthesis

The random-effects meta-analysis was likely to be the most appropriate method to pool data together as some degree of clinical heterogeneity was expected. When studies did not provide sufficient data for use in the meta-analysis, the results were reported separately. If it was deemed that the data were too statistically heterogeneous to combine statistically, a narrative synthesis was utilized.

### Subgroup analysis

Subgroup analyses were planned for the outcomes of depression and anxiety with respect to:

1. studies that included patients following MI +/− non-surgical revascularization compared with studies including patients following CABG, and
2. studies that utilized block randomization (patient-partner dyads randomized to a particular intervention in blocks) compared with studies that randomized sequentially.

### Sensitivity analysis

Sensitivity analyses were conducted for the outcomes of depression and anxiety, specifically analysis which only included studies with partners.

### Other analysis

An analysis was conducted to compare patient only psychological interventions with patient and partner psychological interventions.

### Quality of the evidence

The overall quality of the evidence was assessed using GRADE criteria (risk of bias, consistency, imprecision, indirectness, and publication bias) [17], and was rated as high, moderate or low. Results

### Results of the search

**Figure 1:**
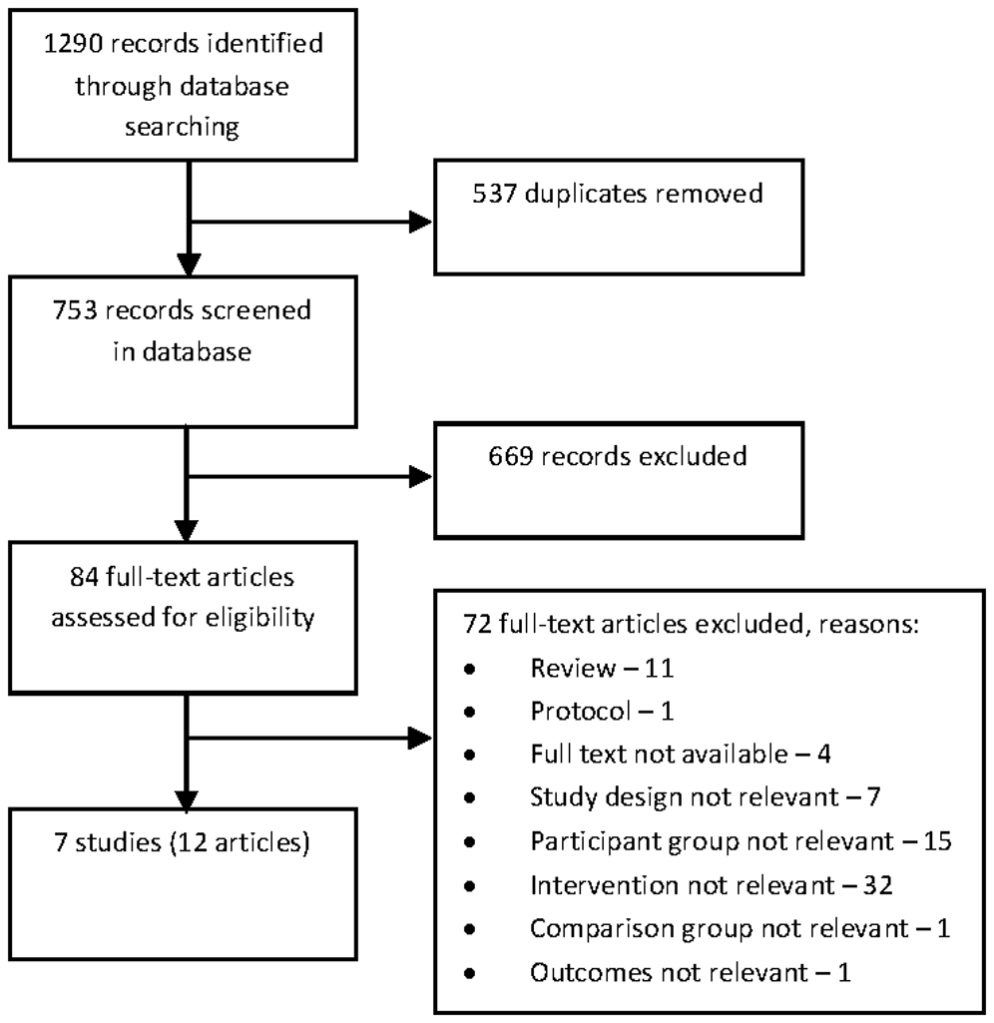
Flow chart showing selection of studies.

Searches of the electronic databases resulted in 1290 records, 537 of these were duplicates, resulting in 753 references in total. Titles and abstracts were screened and 84 records were retrieved and reviewed in full text. Of these full text articles, seven studies (12 articles [18]–[29]) fulfilled the inclusion criteria. The WHO ICTRP search did not identify any additional studies. The seven eligible studies resulted in 673 dyads (patient and partner) randomized. A flowchart of the selection of studies for inclusion is presented in Figure 1.

### Description of studies

All included studies were parallel group randomized controlled trials: four utilized simple randomization [18], [21], [23], [24] and three randomized the treatment groups in blocks to prevent contamination [19], [22], [25]. Studies were conducted in the USA [18], [19], [23], Canada [21], the United Kingdom [22], [25] and Germany [24]. Two studies were conducted in the 1980s [18], [25], two in the 1990s [21], [22] and three did not report when they were conducted [19], [23], [24]. Sample sizes ranged from 42 to 180 patients randomized, mean age ranged from 50.9 to 62.9 years and percentage of male patients ranged from 65 to 100%. Three studies included patients post-MI [18], [22], [25]; two post-MI with or without revascularization [19], [24]; and two post CABG [21], [23]. Duration of follow-up ranged from two to 13 months (Table 1).

**Table 1:** Description of studies.

Author, year	Country conducted	When conducted	Setting	Study design	Patients randomized	Mean age in years (SD)	Male	Diagnosis	Follow-up (mos)
Burgess, 1987 [18]	USA	1981 to 1984	Inpatient, post admission	Parallel group	180	50.9 (7.4)	86%	MI	3, 13
Dracup, 1984 [19]	USA	NA	Outpatient CR	Parallel group (groups randomized in blocks)	63	57^a^ (NA)	90%^b^	MI +/− revascularization	2.5, 6
Hartford, 2002 [21]	Canada	1997 to 1998	Inpatient, post-operatively	Parallel group	166	62.9 (8.6)	86%	CABG	0.1, 1, 2
Johnston, 1999 [22]	Scotland	1992 to 1993	Inpatient, post admission	Parallel group (groups randomized in blocks)	117	56.0 (8.5)	65%	MI	0.25, 2, 6, 12
Lenz, 2000 [23]	USA	NA	Inpatient, pre-operatively	Parallel group	45	60.2 (10.6)	71%	CABG	0.1, 0.5, 1, 1.5, 3
Priebe, 2001 [24]	Germany	NA	Outpatient CR	Parallel group	42	55.4 (8.7)	86%	MI +/− revascularization	9
Thompson, 1989 [25]	England	1986	Inpatient, post admission	Parallel group (groups randomized in blocks)	60	54.4 (7.4)	100%	MI	0.1, 1, 3, 6,

CCU = coronary care unit; IG = intervention group; UC = usual care; MI = myocardial infarction; CR = cardiac rehabilitation; NA = not available; CABG = coronary artery bypass grafting.

a also reported as 58 [18];

b also reported as 97% [18].

The psychological interventions comprised a range of components, format, personnel involved, frequency, number of sessions and duration (Table 2). Studies reported on a variety of outcomes (Table 3) and used a number of different questionnaires.

**Table 2:** Intervention characteristics.

Author, year	Significant other	Personnel	Intervention	Conducted	Individual (dyad)/group	Delivery	Commenced	No. of sessions	Frequency of sessions	Session length (min)	Duration (mos)	Comparison group
Burgess, 1987 [18]	Partner +/− work colleague	Nurses	Based on the cognitive behavioural model; particular focus on how assumptions and beliefs about heart attack and recovery could be altered	Mostly at patient's home; worksite	Individual (dyad)	In person & phone	Last week of hospitalization	NA (mean 6.31)	NA	NA	3	Usual care (conventional hospital rehabilitation)
Dracup, 1984 [19]	Partner	Nurses	Based on symbolic interactionist role theory with stress reduction/relaxation. Group 1: patients and spouses participated Group 2: only patients participated	At the cardiac centre as outpatient	Group	In person	NA	10	Weekly	90	2.5	CR (an hour 3 times weekly for exercise & information – duration NA)
Hartford, 2002 [21]	Partner	Nurses	Information & support	At the hospital as inpatient, then by phone	Individual (dyad)	In person (first), then phone	Hospital discharge	6	On day 1, 2 & 4, week 1, 2 & 7	20–60	1.75	Usual care (details NA)
Johnston, 1999 [22]	Partner	Nurses	Information, counselling & stress management Group 1: as inpatient Group 2: inpatient & outpatient	At the hospital – inpatient/outpatient	Individual (dyad)	In person	Within 3 days of admission	G1: Up to 5 G2: up to 8	NA	NA	1.5	Usual care (details NA)
Lenz, 2000 [23]	Family member (partner, children, siblings, friends)	Cardiac nurses (individual session), researchers (phone sessions) & psychiatric nurses (group session)	Usual care plus videotape for home use, pre-discharge counselling & information session, phone calls, & follow-up dinner with group discussion.	At the hospital by phone	Individual (dyad) & group	In person & phone	Day after discharge	NA	Daily & then bi-weekly	NA	3	Pre-discharge videotape, at least 1 home visit
Priebe, 2001 [24]	Partner	Treating physician & a psychologist	Information, psychoeducation, mainly based on solution focused models	At the CR clinic	Individual (dyad)	In person	Within 12 weeks of event	2 to 4	Approx monthly	60	4	CR (details NA)
Thompson, 1989 [25]	Partner	Nurses	Information, counselling including the couple's reactions to and feelings towards the MI	At the hospital –inpatient	Individual (dyad)	In person	24 hrs post admission	4	Every 1 to 2 days	30	0.1	Usual care (details NA)

CR = cardiac rehabilitation; NA = not available.

**Table 3:** Outcomes measured by included studies.

Author, year	Relevant outcomes – patient	Relevant outcomes – partner
Burgess, 1987 [18]	Depression, anxiety, mortality and social support	
Dracup, 1984, 1985 [19], [20]	Depression, anxiety, smoking, blood pressure, exercise level and weight (body fat)	
Hartford, 2002 [21]	Anxiety	
Johnston, 1999 [22]	Depression, anxiety, functional limitations, mortality, knowledge and satisfaction with treatment	Depression, anxiety, knowledge and satisfaction with treatment
Lenz, 2000 [23]	Depression and satisfaction with treatment	Functional health status
Priebe, 2001 [24]	Depression and health status	
Thompson, 1989–1991 [25]–[29]	Depression, anxiety, mortality, smoking, blood pressure, physical activity, weight, knowledge and satisfaction with treatment	Depression, anxiety, knowledge and satisfaction with treatment

### Risk of bias of included studies

Reporting of methodology used in the included studies was generally poor. Table 4 shows the results of the risk of bias assessment.

**Table 4:** Risk of bias.

Author, year	Random sequence generation	Allocation concealment	Blinding of participants and personnel	Blinding of outcome assessment	Incomplete outcome data	Selective reporting	Other bias
Burgess, 1987 [18]	U	U	NA	U	H	U	NA
Dracup, 1984 [19]	L	H	NA	U	U	U	U
Hartford, 2002 [21]	U	U	NA	L	L	U	NA
Johnston, 1999 [22]	U	H	NA	L	U	U	U
Lenz, 2000 [23]	L	U	NA	U	L	U	NA
Priebe, 2001 [24]	U	U	NA	L	L	U	U
Thompson, 1989 [25]	U	H	NA	U	L	U	U

U = unclear; NA = not applicable; H = high; L = low.

### Effect of intervention

Unpublished data were obtained from the authors of four studies [18], [22], [24], [25].

### Primary outcomes – patient

#### Depressive symptoms

**Figure 2:**
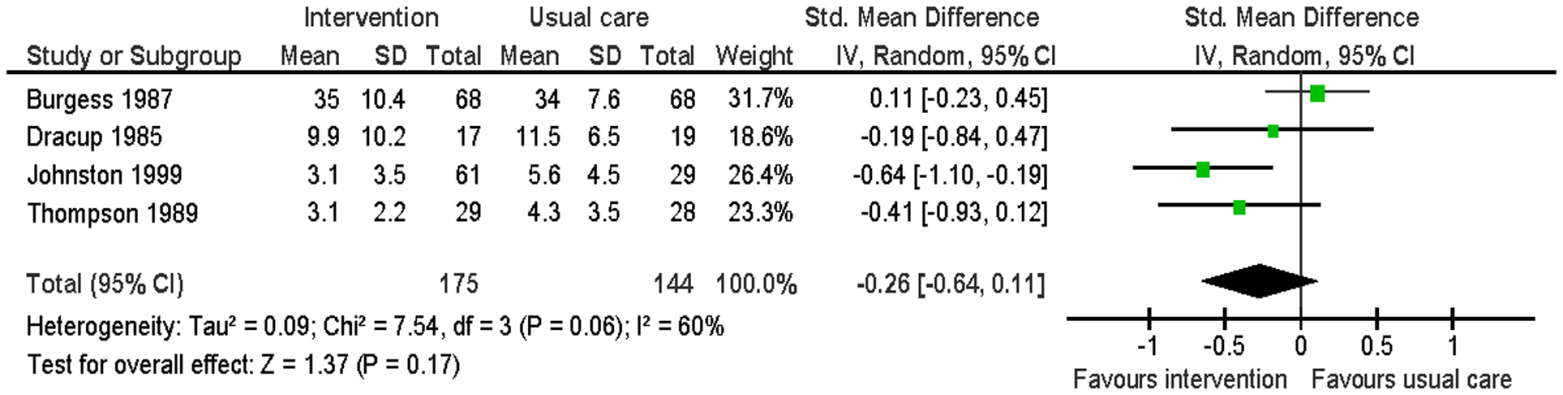
Meta-analysis – outcome: depressive symptoms.

Six studies evaluated this outcome [18], [19], [22]–[25]. Data were able to be combined from four of these [18], [19], [22], [25]. Data were pooled from the Zung Depression scale, the Multiple Adjective Affect Checklist (MAAC) and the HADS-D at follow-up times of six to 13 months (Table 5). There was no significant difference in depressive symptoms between the intervention group and the usual care group (SMD −0.26; 95% CI: −0.64 to 0.11; p = 0.17; n = 319; I^2^ = 60%) (Figure 2). Of the studies which did not report data suitable for meta-analysis, one reported that the usual care group had lower levels of depressive symptoms at follow-up but these differences were non-significant [23], the other reported that the intervention group showed more favourable changes in depressive symptoms than the usual care group [24].

**Table 5:** Outcomes: primary outcomes and tools used.

Author, year	Last follow-up (months)	Depression	Anxiety	Quality of life
Burgess, 1987 [18]	13	Zung Depression scale	Taylor Manifest Anxiety Survey	
Dracup, 1984 [19]	6	Multiple Adjective Affect checklist	Multiple Adjective Affect checklist	
Hartford, 2002 [21]	2		Beck Anxiety Inventory	
Johnston, 1999 [22]	12	HADS-D	HADS-A	Functional Limitations Profile (total)
Lenz, 2000 [23]	3	Center for Epidemiologic Studies-Depression Scale		COOP Functional health status (overall)
Priebe, 2001 [24]	9	HAM-D		Aitken VAS
Thompson, 1989 [25]	6	HADS-D	HADS-A	

HADS-D = Hospital Anxiety and Depression Scale (depression subscale); HAM-D = Hamilton Rating Scale for Depression; VAS = visual analogue scale.

In the above meta-analysis, I^2^ was <50% indicating the presence of heterogeneity. Examining the forest plot (Figure 2) indicated that one study [18] had a different effect size than the others. This study included family members and co-workers and was therefore fundamentally different to the other three studies which only included partners. Repeating the meta-analysis without this study resulted in moderate significantly lower levels of depression in the intervention group compared to the usual care group (SMD −0.46; 95% CI −0.76 to −0.16; p<0.01; I^2^ = 0%).

#### Anxiety

**Figure 3:**
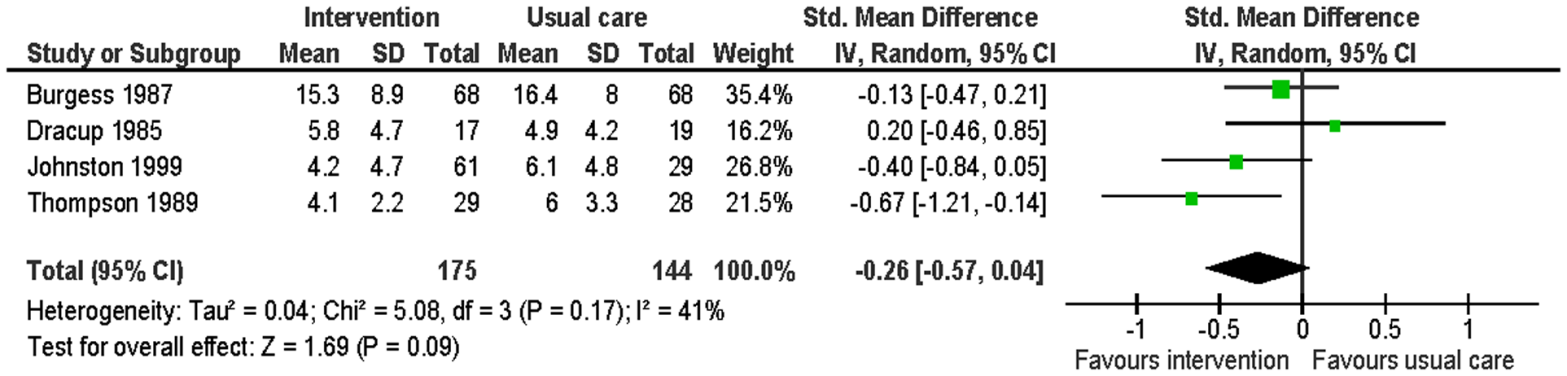
Meta-analysis – outcome: anxiety.

Five studies evaluated this [18], [19], [21], [22], [25]. Data were able to be combined from four of them [18], [19], [22], [25]. Data were pooled from the Taylor Manifest Anxiety Survey, the MAAC and the HADS-A at follow-up times of six to 13 months (Table 5). Participants in the intervention group had non-significantly lower levels of anxiety than the control group at follow-up (SMD −0.26; 95% CI: −0.57 to 0.04; p = 0.09, n = 319, I^2^ = 41%) (Figure 3). The study which did not report suitable data for meta-analysis found no significant effects for group [21].

#### Health-related quality of life

Three studies evaluated this [22]–[24]. Two studies used tools to measure functional status [22], [23] and one study used a tool to measure overall health [24] (Table 5) at follow-up times of six to 13 months. Data were only available from one study (n = 88) [22]. This study found that the intervention group had significantly lower levels of disability than the usual care group at 12 month follow-up (MD −7.40; 95% CI: −12.88 to −1.92; p<0.01). Of the studies which did not provide data, one study (n = 45) reported small non-significant lower levels of disability in the intervention group compare with the usual care group at three months follow-up [23]; the other study (n = 42) reported change scores only – these were significantly lower in the intervention group than the usual care group at nine months follow-up [24].

### Secondary outcomes – patient

#### Mortality

**Figure 4:**
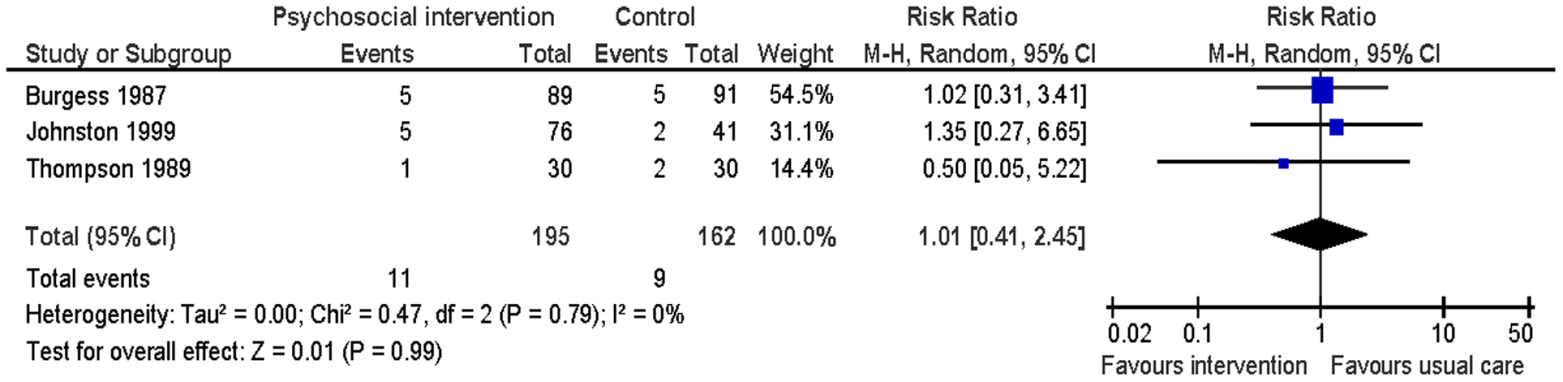
Meta-analysis – outcome: all-cause mortality.

Three studies evaluated this [18], [22], [25] at six to 13 months follow-up. There was no significant difference found between the two groups in all-cause mortality (RR 1.01: 95% CI: 0.41 to 2.45; p = 0.99; n = 357, I^2^ = 0%) (Figure 4).

#### Cardiovascular morbidity (MI, stroke, revascularisations (PCI/CABG))

No studies evaluated this.

#### Cardiovascular risk factors

**Figure 5:**
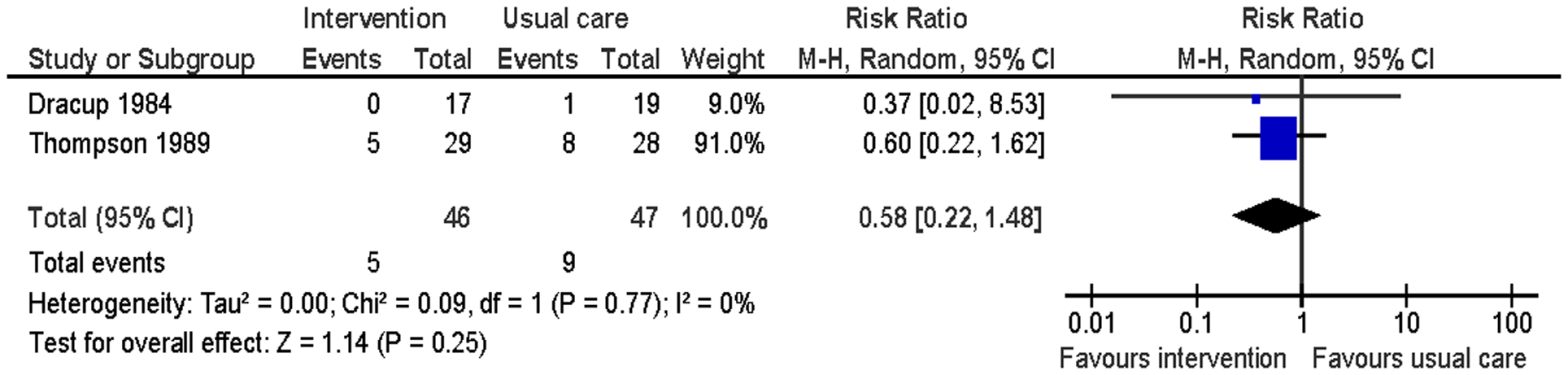
Meta-analysis – outcome: smoking.

**Figure 6:**
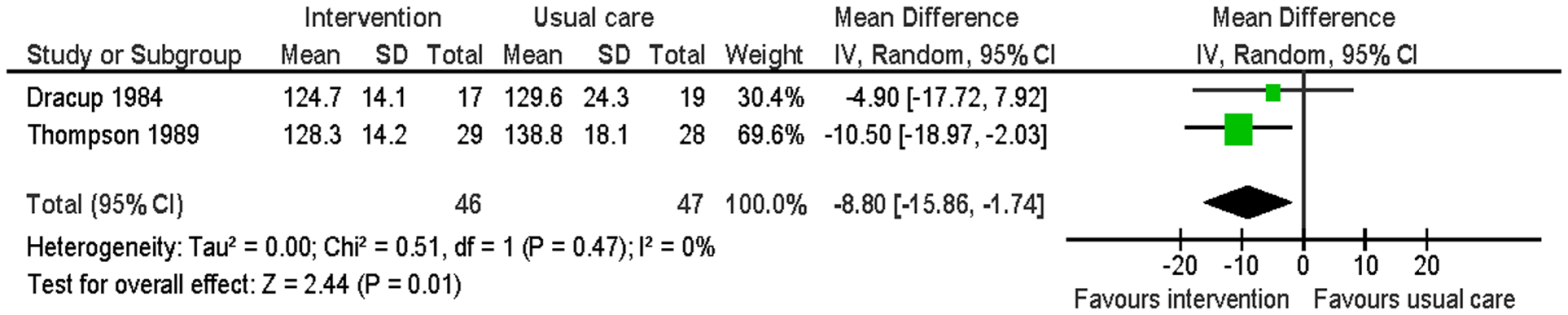
Meta-analysis – outcome: systolic blood pressure.

**Figure 7:**
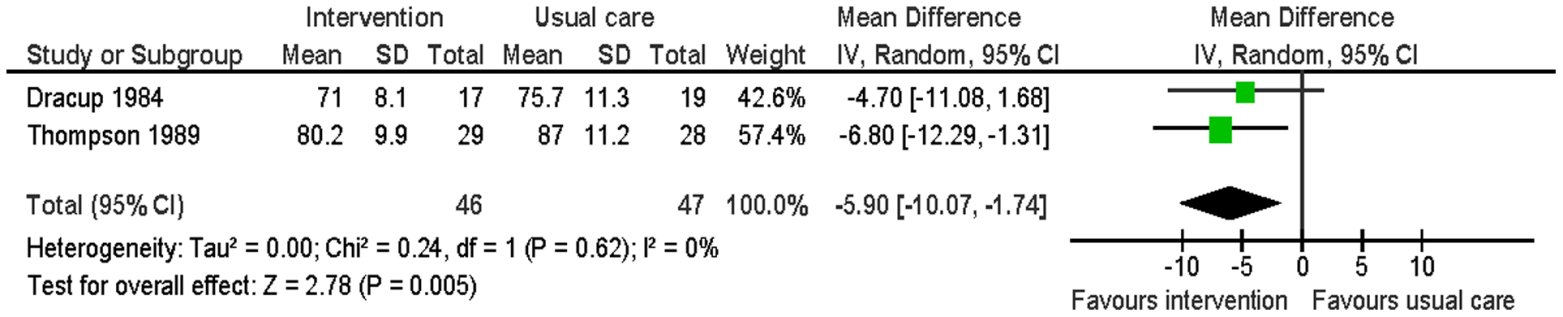
Meta-analysis – outcome: diastolic blood pressure.

**Figure 8:**
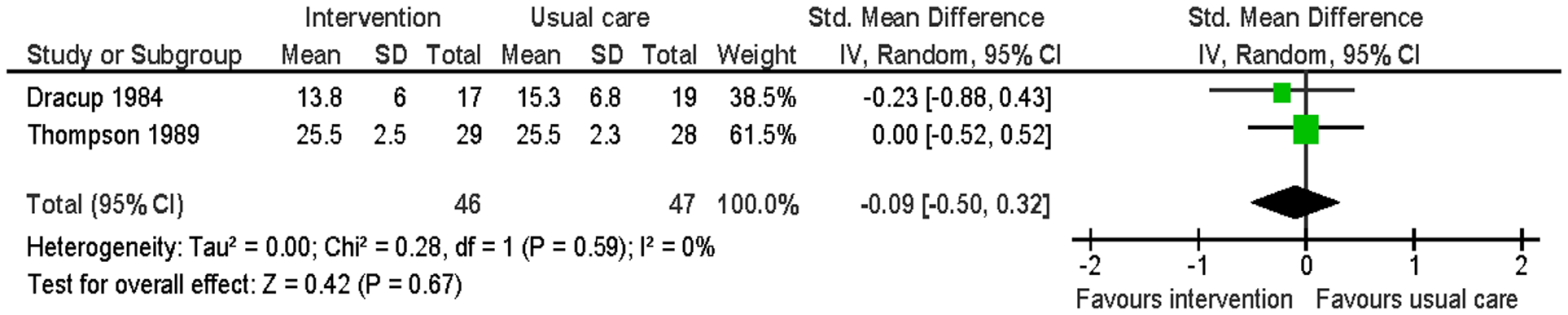
Meta-analysis – outcome: weight.

Two studies reported smoking, blood pressure, physical activity and weight at six months follow-up [19], [25]. There was no significant difference in smoking between the intervention group and the usual care group (RR 0.59: 95% CI: 0.22 to 1.48: p = 0.25; n = 93; I^2^ = 0%) (Figure 5). The intervention group had significantly lower systolic blood pressure (MD −8.80; 95% CI: −15.86 to −1.74; p = 0.01; n = 93; I^2^ = 0%) and diastolic blood pressure (MD −5.90; 95% CI: −10.07 to −1.74; p<0.01; n = 93; I^2^ = 0%) than the usual care group (Figures 6 and 7). For the outcome of physical activity, the data were not combined in a meta-analysis due to significant heterogeneity (I^2^ = 67%, p = 0.08). One study (n = 36) reported that the intervention group had significantly more hours of exercise per week than the usual care group (MD 0.85; 95% CI 0.04 to 1.66; p = 0.04) [19]. The other study (n = 57) reported activity by asking patients to compare their present level of general activity to the level prior to the heart attack on a visual analogue scale from 0 (definitely worse) to 100 (definitely better); there was no significant difference in ratings of general activity between the intervention group and the usual care group (MD 3.6; 95% CI: −10.5 to 17.52; p = 0.61) [25]. For the outcome of weight, this outcome was measured by body mass index by one study [25] and triceps skinfold measurement by the other study [19]. There was no significant difference in weight between the intervention group and the usual care group (SMD −0.09; 95% CI: −0.50 to 0.32; p = 0.67; n = 93; I^2^ = 0%) (Figure 8).

#### Social support

One study evaluated this (n = 136) using a revised version of the questionnaire developed by Caplan and Cobb at 13 months follow-up [18]; there was no significant difference between the intervention and usual care groups (MD 0.0; 95% CI: −0.84 to 0.84; p = 1.0).

#### Knowledge of disease and treatment

**Figure 9:**
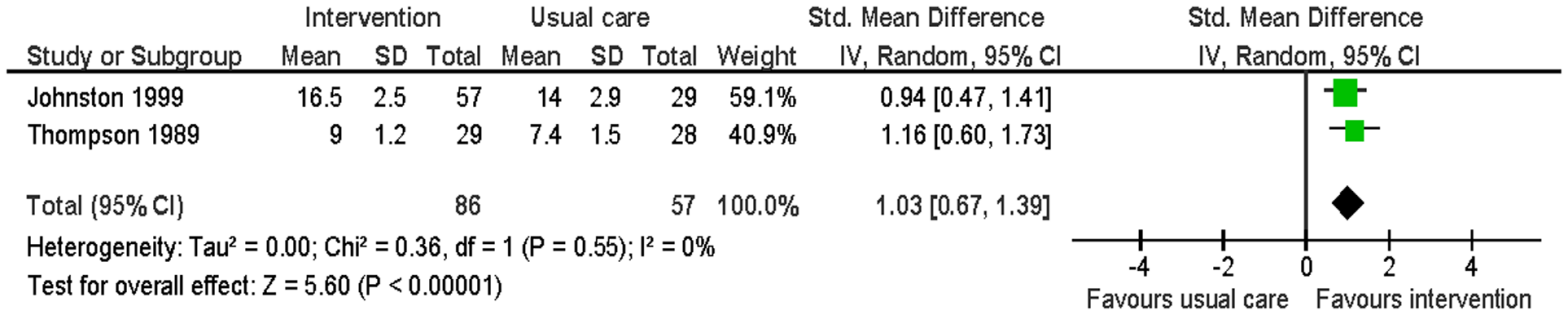
Meta-analysis – outcome: knowledge.

Two studies evaluated this [22], [25]. Knowledge regarding MI, its treatment, and resumption of normal activities was assessed using a 0 to 19 and a 0 to 12 scale at two months and six months. The intervention group had significantly higher knowledge scores than the usual care group (SMD 1.03; 95% CI: 0.67 to 1.39; p<0.01; n = 143; I^2^ = 0%) (Figure 9).

#### Satisfaction with care

Three studies evaluated this [22], [23], [28]. One study (n = 57) measured satisfaction on a 0 to 100 visual analogue scale at six months follow-up. This study found that the intervention group had significantly higher levels of satisfaction with care received than the usual care group (MD 6.30; 95% CI: 1.78 to 10.82; p<0.01) [28].

Of the studies which did not provide data, one (n = 90), with an inpatient only group and an extended group, reported that at two months follow-up the extended group had higher levels of satisfaction with the advice received after the heart attack than the inpatient group, which in turn had higher levels of satisfaction than the usual care group; however only the difference between the extended group and the usual care group was significant [22]. Another study (n = 45) reported higher levels of satisfaction with nursing care in the intervention group than the usual care group at three months follow-up [23].

### Primary outcomes – partner

#### Depression

**Figure 10:**
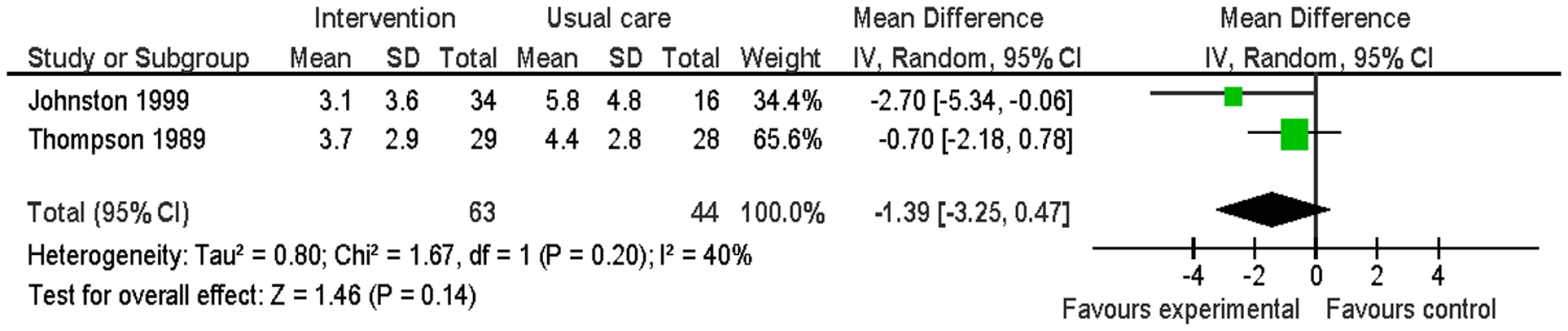
Meta-analysis – outcome: partners' depressive symptoms.

Three studies evaluated this [22], [23], [25]. Data from two of the studies could be combined in the meta-analysis. Depressive symptoms were measured using the HADS-A at six and 12 months. There was no significant difference in depressive symptoms between the intervention group and the usual care group (MD −1.39; 95% CI −3.25 to 0.47; p = 0.20; n = 107; I^2^ = 40%) (Figure 10).

The study which did not provide data suitable for the meta-analysis, reported there was no significant difference in depression symptoms between the intervention group and the control group at three months follow-up [23].

#### Anxiety

**Figure 11:**
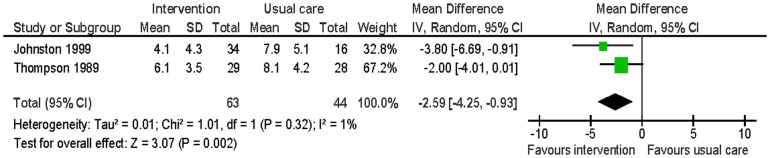
Meta-analysis – outcome: partners' anxiety.

Two studies evaluated this [22], [25]. Anxiety was measured using the HADS-A at six and 12 months. The intervention group had significantly lower levels of anxiety than the usual care group (MD −2.59; 95% CI: −4.25 to −0.93; p<0.01; n = 107; I^2^ = 0%) (Figure 11).

#### Health-related quality of life

One study evaluated this [23]. This study (n = 45) used a tool to measure functional status and found that the intervention group had small non-significant lower levels of disability than the usual care group at three months follow-up.

### Secondary outcomes – partner

#### Knowledge of disease and treatment

**Figure 12:**
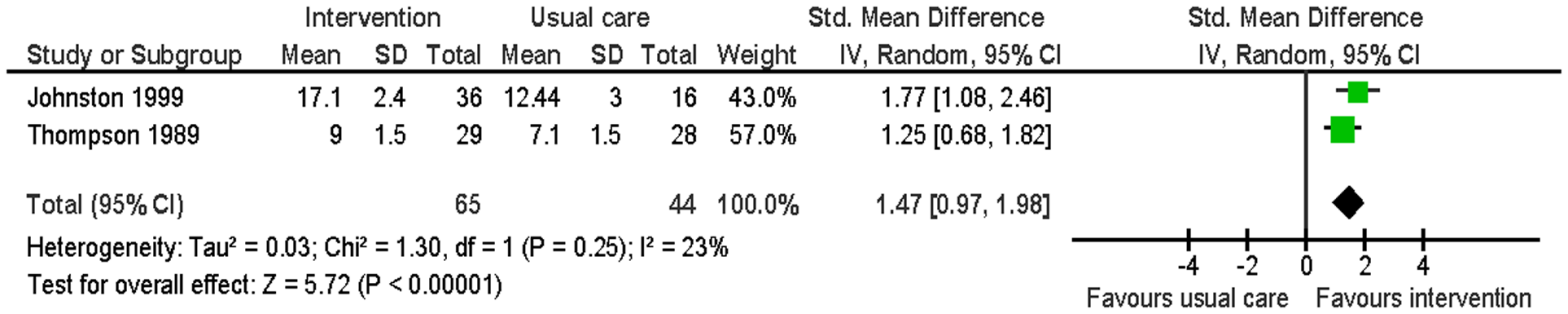
Meta-analysis – outcome: partners' knowledge.

Two studies evaluated this [22], [26]. Knowledge regarding MI, its treatment, and resumption of normal activities was assessed using a 0 to 19 and a 0 to 12 scale at two months and six months. The intervention had significantly high knowledge scores than the usual care group (SMD 1.47; 95% CI: 0.97 to 1.98; p<0.01; n = 109; I^2^ = 23%) (Figure 12).

#### Satisfaction with care

Two studies evaluated this [22], [28]. One study (n = 57) measured satisfaction using a 0 to 100 visual analogue scale at six months follow-up. This study found that the intervention group had significantly higher levels of satisfaction with care received than the usual care group at six months follow-up (MD 4.90; 95% CI: 2.58 to 7.22; p<0.01); [28]. Similarly, the other study (n = 52) which measured this outcome at two months follow-up, found that the intervention group had higher levels of satisfaction with care than the usual care group [22].

### Subgroup analysis

The planned analyses to evaluate the effect of the intervention with regards to: patient type (MI+/− non-surgical revascularization versus CABG); and randomization in blocks compared with individual randomization, was not undertaken due to the small numbers of studies which were included in the meta-analyses [15].

### Sensitivity analysis

#### Depressive symptoms

Excluding the studies with co-workers resulted in moderate significantly lower levels of depressive symptoms in the intervention group compared with the usual care group, as detailed above.

#### Anxiety

Excluding the studies with co-workers did not significantly affect the result for this outcome.

### Patient and partner intervention compared with patient only intervention

One study had two intervention groups – one group included both patient and partner, and one group which only included the patient. No significant differences were seen between the two groups with regards to the outcomes of depressive symptoms (MD −0.30; 95% CI −5.76 to 5.16; p = 0.91; n = 39) and anxiety (MD −0.70; 95% CI: −3.62 to 2.22; p = 0.64; n = 39) at six months follow-up.

### Quality of the evidence

The overall quality of the evidence was low, ‘our confidence in the effect estimate is limited: the true effect may be substantially different from the estimate of the effect’, using the GRADE criteria [17]. This was due to generally poorly reported methodology, uncertainty of publication bias, small imprecise effect size and indirectness. Indirectness related to differences in population, interventions and comparison: the interventions utilised in this review were not a one size fits all – they were dependent by the provider, and the relationship between the provider and the patient-partner dyad.

## Discussion

### Summary of findings

This systematic review found that psychological interventions for patient and partner improves health-related quality of life, blood pressure, knowledge, and satisfaction with care for patients, and anxiety, knowledge, and satisfaction with care for partners. There was a non-significant trend for improvements in anxiety for patients, and depressive symptoms for both patient and partner. There was no evidence of a statistically significant effect on mortality, morbidity or other cardiovascular risk factors for patients, or social support for patients and partners. For the outcome of depressive symptoms, there was significant statistical heterogeneity due to a study which included co-workers and which aimed to improve return to work rates. When this study was removed from the analysis the level of statistical heterogeneity reduced and the effect size increased. This would suggest that including co-workers does not provide benefit and appears to be detrimental. Excluding this study from the meta-analysis was deemed appropriate as the intervention in this study was fundamentally different to the other studies.

### Strengths and limitations

This is the first systematic review of psychological interventions for patients with CHD and their partners. This review was well-conducted utilising Cochrane methodology. The overall quality of evidence was low, however. This was due to potential risk of bias of the included studies, potential for publication bias, imprecise effect size and uncertain external validity.

There are a number of factors which are likely to have affected external validity. Firstly, the studies included were undertaken at least a decade ago, when standard cardiovascular management and aftercare, including rehabilitation, would have been less sophisticated than today. Secondly, interventions utilised in this review were not a one size fits all – they were dependent to a large extent on the provider, and the relationship between the provider and the patient-partner dyad.

### Findings in context with previous studies

Despite the limitations, these findings lend some support to, and are consistent with, previous systematic reviews which evaluated, and found benefit of, patient-partner interventions for patients with a range of chronic diseases [12], [13]. A systematic review which reported on mortality for patients with chronic diseases, found that patient and partner interventions results in improved mortality [13], but this earlier review included more studies and patients and thus had great power.

Interestingly, partners were generally found to have higher levels of depressive symptoms and anxiety than patients. This is consistent with another systematic review which assessed the effect of a cardiac event on the partners [8]. This finding suggests that partners are more adversely affected by the cardiac event than the patient. However, these results could be confounded by gender – there are more women in the partner group and women tend to have higher levels of depressive symptoms and anxiety than men [30].

As with other reviews [11], psychological interventions were heterogeneous in terms of model of intervention, personnel, format (group or individual, phone or in person), number of sessions and duration. However, with the exception of the study which included co-workers, the interventions appear to result in similar effect.

### Effectiveness of patient-partner psychological interventions compared with patient only interventions

Are we able to conclude from the results of this review, that patient and partner psychological interventions are more (or less) effective than psychological interventions which only include patients? One of the studies included in this review had two intervention groups – one for patients and partners, the other for patients only [19]. No significant differences were found for the outcomes of depressive symptoms or anxiety at six months follow-up. However, this result needs to be interpreted with caution due to the small sample.

Comparing the results of this review with an earlier review [11], which predominately included patient only interventions, it would appear that there are similar benefits of patient and partner inventions. Although this review evaluated the SMD at follow-up, the earlier review change over time [11], the effect sizes are comparable.

In this review, studies were only included if at least 50% of intervention sessions were attended by the partner/family; the other review [11] included studies with minimal partner involvement – the patient's partner was ‘encouraged to attend’, or was included in a part of the intervention. Therefore the partners in the ‘family’ studies of the other review [11] would have significantly less involvement, potentially diluting the effect of partner inclusion. The other review [11] included interventions which were delivered by health care workers with specific training in psychological techniques; this review included interventions which were delivered by all health professionals and did not require them to have specific training.

All of the studies were undertaken at least over a decade ago and standard cardiovascular management and aftercare, including rehabilitation, has probably improved since then. Although there have been many changes in the management of cardiac patients during that period, it was decided to include studies regardless of when they were conducted, though recognizing that usual care may also have changed markedly over that time.

Education was included as a component of some of the interventions; it is difficult, therefore, to quantify the effect of each of the psychological component and educational components. The dose response of the intervention was difficult to measure as most studies did not report actual number of sessions the partner attended.

The extent of involvement of the partner in the usual care group generally was not documented. Including partners in the study, even if the patients (and partners) were randomized to usual care, could lead to partners having more involvement than would normally occur, thus potentially diluting the effect of the intervention. The majority of the psychological interventions were provided by registered nurses and in only one trial by a psychologist. While nurse counselling may not be comparable with psychological therapy, most nurses are well trained in contemporary basic psychological techniques, which are usually sufficient to address the common psychological sequelae exhibited by the vast majority of these patients and partners. Unrecorded use of pharmacotherapy alongside counselling could mask any effect of the latter but the studies reviewed did not document the use of pharmacotherapy.

### Implications of findings

Although the findings do not provide strong evidence attesting to the effectiveness of psychological interventions for patients and partners, they do provide valuable evidence that may inform the design and conduct of new studies aiming to demonstrate such effectiveness.

### Future directions

In order to determine which psychological interventions are most effective, it is recommended that a large, adequately powered, trial assessing psychological interventions for patients alone, compared with psychological interventions for patients and partners, is undertaken. A number of factors could be tested, including the time to commence the intervention, its intensity and duration, but taking into account a clear definition of the intervention, its mode of delivery and content characteristics, as well as the type, training and qualifications of the therapist.

## Conclusion

This systematic review found that psychological interventions for patients with CHD and their partners improves health-related quality of life, blood pressure, knowledge, and satisfaction with care for patients, and anxiety, knowledge, and satisfaction with care for partners. There was a non-significant trend for improvements in anxiety for patients, and depressive symptoms for both patient and partner. However, as the overall quality of the evidence was low, these results should be interpreted with caution.

## Supporting Information

Checklist S1 (DOCX)

## References

[1] Thombs BD, Bass EB, Ford DE, Stewart KJ, Tsilidis KK, et al (2006) Prevalence of depression in survivors of acute myocardial infarction: Review of the evidence. J Gen Intern Med 21: 30–38. 16423120 10.1111/j.1525-1497.2005.00269.x PMC1484630

[2] Roest AM, Martens EJ, Denollet J, De Jonge P (2010) Prognostic association of anxiety post myocardial infarction with mortality and new cardiac events: A meta-analysis. Psychosom Med 72: 563–569. 20410247 10.1097/PSY.0b013e3181dbff97

[3] Barth J, Schumacher M, Herrmann-Lingen C (2004) Depression as a risk factor for mortality in patients with coronary heart disease: A meta-analysis. Psychosom Med 66: 802–813. 15564343 10.1097/01.psy.0000146332.53619.b2

[4] Nicholson A, Kuper H, Hemingway H (2006) Depression as an aetiologic and prognostic factor in coronary heart disease: A meta-analysis of 6362 events among 146 538 participants in 54 observational studies. Eur Heart J 27: 2763–2774. 17082208 10.1093/eurheartj/ehl338

[5] Van Melle JP, De Jonge P, Spijkerman TA, Tijssen JGP, Ormel J, et al (2004) Prognostic association of depression following myocardial infarction with mortality and cardiovascular events: A meta-analysis. Psychosom Med 66: 814–822. 15564344 10.1097/01.psy.0000146294.82810.9c

[6] Baumeister H, Hutter N, Bengel J, Härter M (2011) Quality of life in medically Ill persons with comorbid mental disorders: A systematic review and meta-analysis. Psychother Psychosom 80: 275–286. 21646822 10.1159/000323404

[7] Dickens C, Cherrington A, McGowan L (2012) Depression and health-related quality of life in people with coronary heart disease: a systematic review. Eur J Cardiovasc Nurs 11: 265–275. 22457381 10.1177/1474515111430928

[8] Randall G, Molloy GJ, Steptoe A (2009) The impact of an acute cardiac event on the partners of patients: A systematic review. Health Psychol Rev 3: 1–84.

[9] Baumeister H, Hutter N, Bengel J (2011) Psychological and pharmacological interventions for depression in patients with coronary artery disease. Cochrane Database Syst Rev CD008012. 21901717 10.1002/14651858.CD008012.pub3 PMC7389312

[10] Linden W, Phillips MJ, Leclerc J (2007) Psychological treatment of cardiac patients: A meta-analysis. Eur Heart J 28: 2972–2984. 17984133 10.1093/eurheartj/ehm504

[11] Whalley B, Rees K, Davies P, Bennett P, Ebrahim S, et al (2011) Psychological interventions for coronary heart disease. Cochrane Database Syst Rev CD002902. 21833943 10.1002/14651858.CD002902.pub3

[12] Hartmann M, Bäzner E, Wild B, Eisler I, Herzog W (2010) Effects of interventions involving the family in the treatment of adult patients with chronic physical diseases: A meta-analysis. Psychother Psychosom 79: 136–148. 20185970 10.1159/000286958

[13] Martire LM, Lustig AP, Schulz R, Miller GE, Helgeson VS (2004) Is it beneficial to involve a family member? A meta-analysis of psychosocial interventions for chronic illness. Health Psychol 23: 599–611. 15546228 10.1037/0278-6133.23.6.599

[14] Martire LM, Schulz R, Helgeson VS, Small BJ, Saghafi EM (2010) Review and meta-analysis of couple-oriented interventions for chronic illness. Ann Behav Med 2010 40: 325–342. 10.1007/s12160-010-9216-2 PMC4101802 20697859

[15] Higgins JPT, Green S (2011) Cochrane Handbook for Systematic Reviews of Interventions Version 5.1. The Cochrane Collaboration

[16] Review Manager (RevMan) (2011) Version 5.1. The Cochrane Collaboration

[17] Balshem H, Helfand M, Schünemann HJ, Oxman AD, Kunz R, et al (2011) GRADE guidelines: 3. Rating the evidence. J Clin Epidemiol 64: 401–406. 21208779 10.1016/j.jclinepi.2010.07.015

[18] Burgess AW, Lerner DJ, D'Agostino RB, Vokonas PS, Hartman CR, et al (1987) A randomized control trial of cardiac rehabilitation. Soc Sci Med 24: 359–370. 3551088 10.1016/0277-9536(87)90154-7

[19] Dracup K, Meleis AI, Clark S, Clyburn A, Shields L, et al (1984) Group counseling in cardiac rehabilitation: effect on patient compliance. Patient Educ Couns 6: 169–177. 10269521 10.1016/0738-3991(84)90053-3

[20] Dracup K (1985) A controlled trial of couples group counseling in cardiac rehabilitation. J Cardiopulm Rehabil 5: 436–442.

[21] Hartford K, Wong C, Zakaria D (2002) Randomized controlled trial of a telephone intervention by nurses to provide information and support to patients and their partners after elective coronary artery bypass graft surgery: effects of anxiety. Heart Lung 31: 199–206. 12011810 10.1067/mhl.2002.122942

[22] Johnston M, Foulkes J, Johnston DW, Pollard B, Gudmundsdottir H (1999) Impact on patients and partners of inpatient and extended cardiac counseling and rehabilitation: A controlled trial. Psychosom Med 61: 225–233. 10204976 10.1097/00006842-199903000-00015

[23] Lenz ER, Perkins S (2000) Coronary artery bypass graft surgery patients and their family member caregivers: outcomes of a family-focused staged psychoeducational intervention. Appl Nurs Res 13: 142–150. 10960998 10.1053/apnr.2000.7655

[24] Priebe S, Sinning U (2001) Effects of a brief couples therapy intervention in coronary rehabilitation. A controlled study. Psychother Psychosom Med Psychol 51: 276–280. 11496446 10.1055/s-2001-15621

[25] Thompson DR (1989) A randomized controlled trial of in-hospital nursing support for first time myocardial infarction patients and their partners: effects on anxiety and depression. J Adv Nurs 14: 291–297. 2738227 10.1111/j.1365-2648.1989.tb03416.x

[26] Thompson DR (1991) Effect of in-hospital counseling on knowledge in myocardial infarction patients and spouses. Patient Educ Couns 18: 171–177.

[27] Thompson DR, Meddis R (1990) Wives' responses to counselling early after myocardial infarction. J Psychosom Res 34: 249–258. 2341994 10.1016/0022-3999(90)90081-e

[28] Thompson DR, Webster RA, Meddis R (1990) In-hospital counselling for first-time myocardial infarction patients and spouses: effects on satisfaction. J Adv Nurs 15: 1064–1069. 2229705 10.1111/j.1365-2648.1990.tb01987.x

[29] Thompson DR, Meddis R (1990) A prospective evaluation of in-hospital counselling for first time myocardial infarction men. J Psychosom Res 34: 237–248. 2341993 10.1016/0022-3999(90)90080-n

[30] Leach LS, Christensen H, Mackinnon AJ, Windsor TD, Butterworth P (2008) Gender differences in depression and anxiety across the adult lifespan: The role of psychosocial mediators. Soc Psychiatry Psychiatr Epidemiol 43: 983–998. 18575787 10.1007/s00127-008-0388-z

