# Correction: Psychological Interventions for Patients with Coronary Heart Disease and Their Partners: A Systematic Review

**DOI:** 10.1371/annotation/79b46b67-786c-47cf-945e-915906ac2e55

**Published:** 2013-10-28

**Authors:** Jane Reid, Chantal F. Ski, David R. Thompson

This article was republished on October 23, 2013, because an incorrect version of the article was originally published. Please download this article again to view the correct version. 

