# Supplementary material for: Psychological Interventions for Patients with Coronary Heart Disease and Their Partners: A Systematic Review
Source: PLoS One. 2013 Sep 5;8(9):e73459. doi: 10.1371/journal.pone.0073459 (PMC3764157; doi:10.1371/journal.pone.0073459)
Supplement: Checklist S1 — (DOCX) [file pone.0073459.s001.docx]

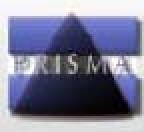
**PRISMA 2009 Checklist**

| **Section/topic** | **#** | **Checklist item** | **Reported** |  |  |
| --- | --- | --- | --- | --- | --- |
|  |  |  | **on page #** |  |  |
|  |  |  |  |  |  |
|  |  |  |  |  |  |
| **TITLE** |  |  |  |  |  |
| Title | 1 | Identify the report as a systematic review, meta-analysis, or both. | 1 Title page |  |  |
|  |  |  |  |  |  |
| **ABSTRACT** |  |  |  |  |  |
| Structured summary | 2 | Provide a structured summary including, as applicable: background; objectives; data sources; study eligibility criteria, | 2 Abstract |  |  |
|  |  | participants, and interventions; study appraisal and synthesis methods; results; limitations; conclusions and |  |  |  |
|  |  | implications of key findings; systematic review registration number. |  |  |  |
|  |  |  |  |  |  |
| **INTRODUCTION** |  |  |  |  |  |
| Rationale | 3 | Describe the rationale for the review in the context of what is already known. | 4-5 Intro |  |  |
|  |  |  |  |  |  |
| Objectives | 4 | Provide an explicit statement of questions being addressed with reference to participants, interventions, comparisons, | 4-5 Intro |  |  |
|  |  | outcomes, and study design (PICOS). |  |  |  |
|  |  |  |  |  |  |
| **METHODS** |  |  |  |  |  |
| Protocol and registration | 5 | Indicate if a review protocol exists, if and where it can be accessed (e.g., Web address), and, if available, provide | No |  |  |
|  |  | registration information including registration number. |  |  |  |
|  |  |  |  |  |  |
| Eligibility criteria | 6 | Specify study characteristics (e.g., PICOS, length of follow‐up) and report characteristics (e.g., years considered, | 5-7 Methods |  |  |
|  |  | language, publication status) used as criteria for eligibility, giving rationale. |  |  |  |
|  |  |  |  |  |  |
| Information sources | 7 | Describe all information sources (e.g., databases with dates of coverage, contact with study authors to identify | 7-8 Methods |  |  |
|  |  | additional studies) in the search and date last searched. |  |  |  |
|  |  |  |  |  |  |
| Search | 8 | Present full electronic search strategy for at least one database, including any limits used, such that it could be | 7-8 Methods |  |  |
|  |  | repeated. |  |  |  |
|  |  |  |  |  |  |
| Study selection | 9 | State the process for selecting studies (i.e., screening, eligibility, included in systematic review, and, if applicable, | 8-9 Methods |  |  |
|  |  | included in the meta‐analysis). |  |  |  |
|  |  |  |  |  |  |
| Data collection process | 10 | Describe method of data extraction from reports (e.g., piloted forms, independently, in duplicate) and any processes | 8-9 Methods |  |  |
|  |  | for obtaining and confirming data from investigators. |  |  |  |
|  |  |  |  |  |  |
| Data items | 11 | List and define all variables for which data were sought (e.g., PICOS, funding sources) and any assumptions and | 8-10 Methods |  |  |
|  |  | simplifications made. |  |  |  |
|  |  |  |  |  |  |
| Risk of bias in individual | 12 | Describe methods used for assessing risk of bias of individual studies (including specification of whether this was | 9-10 Methods |  |  |
| studies |  | done at the study or outcome level), and how this information is to be used in any data synthesis. |  |  |  |
|  |  |  |  |  |  |
| Summary measures | 13 | State the principal summary measures (e.g., risk ratio, difference in means). | 9-11 Methods |  |  |
|  |  |  |  |  |  |
| Synthesis of results | 14 | Describe the methods of handling data and combining results of studies, if done, including measures of consistency | 11-17 Methods |  |  |
|  |  | (e.g., I^2^) for each meta‐analysis. |  |  |  |


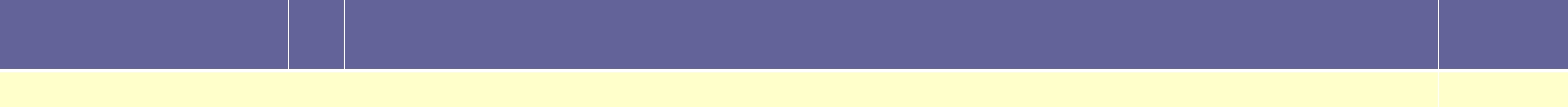


Page 1 of 2


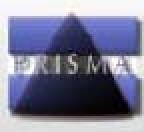
**PRISMA 2009 Checklist**

| **Section/topic** | **#** | **Checklist item** | **Reported** |  |  |
| --- | --- | --- | --- | --- | --- |
|  |  |  | **on page #** |  |  |
|  |  |  |  |  |  |
|  |  |  |  |  |  |
| Risk of bias across studies | 15 | Specify any assessment of risk of bias that may affect the cumulative evidence (e.g., publication bias, selective | 13, 19-20 |  |  |
|  |  | reporting within studies). | Methods |  |  |
|  |  |  |  |  |  |
| Additional analyses | 16 | Describe methods of additional analyses (e.g., sensitivity or subgroup analyses, meta-regression), if done, indicating | 19 Methods |  |  |
|  |  | which were pre‐specified. |  |  |  |
|  |  |  |  |  |  |
| **RESULTS** |  |  |  |  |  |
| Study selection | 17 | Give numbers of studies screened, assessed for eligibility, and included in the review, with reasons for exclusions at | Fig 1 PRISMA |  |  |
|  |  | each stage, ideally with a flow diagram. | Results |  |  |
|  |  |  |  |  |  |
| Study characteristics | 18 | For each study, present characteristics for which data were extracted (e.g., study size, PICOS, follow-up period) and | Tables 1-3 |  |  |
|  |  | provide the citations. | Results |  |  |
|  |  |  |  |  |  |
| Risk of bias within studies | 19 | Present data on risk of bias of each study and, if available, any outcome level assessment (see item 12). | Table 4 Results |  |  |
|  |  |  |  |  |  |
| Results of individual studies | 20 | For all outcomes considered (benefits or harms), present, for each study: (a) simple summary data for each | Figs 2-12 Results |  |  |
|  |  | intervention group (b) effect estimates and confidence intervals, ideally with a forest plot. |  |  |  |
|  |  |  |  |  |  |
| Synthesis of results | 21 | Present results of each meta-analysis done, including confidence intervals and measures of consistency. | Figs 2-12 Results |  |  |
|  |  |  |  |  |  |
| Risk of bias across studies | 22 | Present results of any assessment of risk of bias across studies (see Item 15). | Table 4 Results |  |  |
|  |  |  |  |  |  |
| Additional analysis | 23 | Give results of additional analyses, if done (e.g., sensitivity or subgroup analyses, meta-regression [see Item 16]). | 19 Results |  |  |
|  |  |  |  |  |  |
| **DISCUSSION** |  |  |  |  |  |
| Summary of evidence | 24 | Summarize the main findings including the strength of evidence for each main outcome; consider their relevance to | 20 Discussion |  |  |
|  |  | key groups (e.g., healthcare providers, users, and policy makers). |  |  |  |
|  |  |  |  |  |  |
| Limitations | 25 | Discuss limitations at study and outcome level (e.g., risk of bias), and at review-level (e.g., incomplete retrieval of | 21 Discussion |  |  |
|  |  | identified research, reporting bias). |  |  |  |
|  |  |  |  |  |  |
| Conclusions | 26 | Provide a general interpretation of the results in the context of other evidence, and implications for future research. | 21-24 Conclusions |  |  |
|  |  |  |  |  |  |
| **FUNDING** |  |  |  |  |  |
| Funding | 27 | Describe sources of funding for the systematic review and other support (e.g., supply of data); role of funders for the | Not funded |  |  |
|  |  | systematic review. |  |  |  |
|  |  |  |  |  |  |


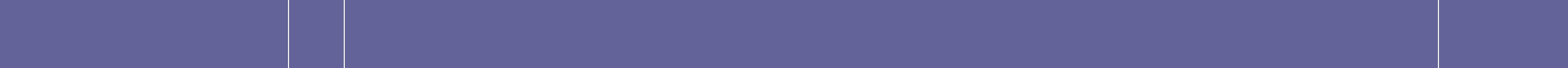


*From:* Moher D, Liberati A, Tetzlaff J, Altman DG, The PRISMA Group (2009). Preferred Reporting Items for Systematic Reviews and Meta-Analyses: The PRISMA Statement. PLoS Med 6(6): e1000097. doi:10.1371/journal.pmed1000097
